# Strayed dogs sentinels of *Trichinella britovi* infection in Kosovo

**DOI:** 10.1051/parasite/2011183281

**Published:** 2011-08-15

**Authors:** S. Watier-Grillot, I. Vallée, S.A. Lacour, A. Cana, B. Davoust, J.L. Marié

**Affiliations:** 1 Direction Régionale du Service de Santé des Armées de Toulon, BP 860, 83800 Toulon Cedex 9, France. French Forces Medical Service, Working Group on Animal Epidemiology, Secteur Vétérinaire de Marseille BP 30182 13276 Marseille Cedex 9 France; 2 French NRL for Food Borne Parasites, JRU BIPAR ANSES, ENVA, UPEC, ANSES Animal Health Laboratory 23, avenue du Général de Gaulle 94706 Maisons-Alfort Cedex France; 3 Veterinarian, private practice Mitrovica Kosovo

Sir,

Trichinellosis is one of the most widespread parasitic zoonoses in the world. Over the past decade, Eastern Europe has experienced a re-emergence of trichinellosis, connected with the social, political and economic changes of the 1990s. A high prevalence of *Trichinella* infection in domestic animals and in humans has been reported in Bulgaria, Serbia and Montenegro, Romania and Croatia (Cuperlovic *et al.*, 2005; Blaga *et al.*, 2007). Between 2008 and 2010, the presence of trichinellosis was reported on animals in Serbia and Montenegro, in Bulgaria. Human cases of trichinellosis were also recently reported in Serbia and Montenegro, Bulgaria, Albania (OIE, WAHID database). Update data concerning *Trichinella* infection in animals and humans in Kosovo are not available. Kosovo is located in the heart of South East Europe, in the middle of the Balkan Peninsula. Kosovo is situated between the 42° and 44° parallels of Northern hemisphere and between the 20° and 22° meridians. Kosovo has common borders with the aforementioned countries of Eastern Europe. To improve the knowledge of the epidemiology of trichinellosis in this area, the detection of *Trichinella* spp. was performed on muscles collected on carcasses of stray dogs from the outskirts of the city of Mitrovica, north Kosovo.

In October 2008, muscle samples were collected from 25 stray dogs from the suburbs of Mitrovica, a city located in the Northern part of Kosovo (19° 36’ 49’’ E; 44° 58’ 56’’ N). They were shot in a landfill situated between the cities of Mitrovica and Zvecan (20° 50’ 23’’ E; 42° 54’ 24’’ N). The dogs included in the study were shot as part of a rabies prevention program, by hunters employed by the civilian authorities of the city. Muscles samples were taken from masseter muscles (about 20% of whole samples) dorsal muscles (about 40% of the samples) and diaphragm muscles and pillars (about 40% of the samples).

Muscle samples were kept frozen at -20 °C until further processing in the French national reference laboratory for Food borne parasite, Maisons-Alfort, France.

*Trichinella* larvae were identified using a multiplex PCR as described by Pozio and la Rosa (Pozio & la Rosa, 2003). All dogs included in the study were crossbreed (hound, sheepdog), medium – weighed and mature (Table I). Sex (male or female) was not recorded at the time of sampling.

**Table I.** Results of the search and identification of *Trichinella* larvae in muscles samples collected on 25 dogs from Mitrovica, north Kosovo.

Dog	Age (years)	Weight (kg)	Larvae/g	Species
1–21			–	–
22			0.9	*T. britovi*
23	2-5	15-40	0.4	*T. britovi*
24			57	*T. britovi*
25			22.8	*T. britovi*

Overall, 16% (4/25) of the dogs were infected by *Trichinella*. *Trichinella britovi* was identified in all the positive samples. This result proves the circulation of the parasite in this area.

In Kosovo, stray dogs live in the outskirts of the cities and villages. Because of this periurban location, they can create a link between wild and domestic animals, as described by Pozio (Pozio, 2007). The area located on north Mitrovica is occupied by forests. Here, stray dogs come into contact with wildlife. Various species of wild animals are reservoir hosts for *T. britovi*, transmitted within a sylvatic cycle (Gottstein *et al.*, 2009). Peroral infection of stray dogs may occur after ingestion of muscle tissue from infectious prey animals (rodents, birds...) or by consumption of infectious tissues from a carrion of homologous (cannibalism) or heterologous species (boars, foxes, wolves, birds, rodents...).

One of the most important biological factors promoting transmission of the parasite is the physiological ability of muscle-stage larvae to survive in decaying carcasses/carrions. Encapsulated larvae of *T. britovi* in mouse carcasses packed in plastic vials have been found to be infective for laboratory animals up to 45 days at room temperature even if the muscle is completely liquefied (Pozio, unpublished data). Moreover, muscle larvae of *Trichinella* spp. can survive even at low freezing temperatures, maintaining infectivity for future hosts. *T. britovi* can survive in frozen carrion for up to one year (Dick & Pozio, 2001). The survival of muscle larvae after freezing occurs mainly when these larvae parasitize striated muscles of carnivores (bears, foxes, dogs...) (Gottstein *et al.*, 2009). These well-established environmental adaptations go towards increasing the survival of the parasite in nature and perpetuating the transmission of the parasite to wildlife and stray dogs.

Stray dogs also go near and even enter in the city of Mitrovica to find out food, especially during cold season (from October to March-April). They eat mainly garbage: rubbish cans from Mitrovica and from the villages around Mitrovica; landfill located near Mitrovica, where all the garbage from the outskirts of Mitrovica are deposited. Garbage are not buried and are stored open-cast in the landfill. They are therefore easily accessible to feral animals. Because Mitrovica is located near a Serb area, where swine herds are bred and pork meats are consumed, garbage may contain pork scraps and even pork carcasses. In several countries of Central Eastern Europe, the transient breakdown of governmental veterinary services and state farms, accompanied by economic problems and war, have resulted in sharp increase in the incidence of *Trichinella* infection among domestic pig herds, with prevalence rates reaching 50% in some villages in 1990s (Murrell & Pozio, 2000). In the neighbouring Serbia, the consequences have been a 300% increase in pig infection and a concomitant large increase of human cases of trichinellosis (Murrell & Pozio, 2000).

Human trichinellosis may be acquired through the consumption of raw or undercooked meat (*e.g.*, pork, horse and game meat) containing infective larvae of *Trichinella* spp. (Gottstein *et al.*, 2009). Immediate cause of recent human outbreaks in Eastern Europe was the consumption of traditionally prepared sausages (Dupouy-Camet, 2006). Pig contamination mainly occurs by eating scraps of infectious meats mixed with food, by eating rodents present in the pig farm or by caudophagy. Horse can also play a part in infection of stray dogs in Kosovo. Kosovo is mostly rural and horses are used as draught animals. Stray dogs can become infected by consumption of carcasses of infected horses. In the past 30 years, horse meat has been identified as the main source of human trichinellosis in Europe, with more than 3,350 cases reported in 14 outbreaks (Pozio *et al.*, 2005; Boireau *et al.*, 2000). Horses fattening with pork scraps before slaughtering or consumption of rodents crushed and mixed with the fodder by accident are sources of contamination of horses (Murrell *et al.*, 2004).

Considering the high parasitic loads revealed on a number of dogs included in the study, stray dogs from the outskirts of Mitrovica probably become infected from several sources of contamination. As they are carnivorous, they concentrate the parasite. The cycle of transmission of *Trichinella* spp. in animals in Kosovo is not precisely known as knowledge is only partial. Further studies would be necessary, including sampling on wild and out-of-town animals (rodents, stray cats...).

In this study, local stray dogs represent bio-indicators for a biological risk. In areas where epidemiological data are unknown or incomplete, local animals can be useful sentinels for humans, for sanitary risks of environmental origin. Because there are a lot of potential reservoir hosts of the parasite, especially among wildlife, *Trichinella* infection cannot be eradicated. Nevertheless the disease can be efficiently prevented in humans by means of individual and public health measures. Improvement of meat control and education of the consumers (to cook potentially infected meat thoroughly) are key preventive measures. Travellers should also be informed of the risks of illegal importations of traditional meat products from Central and Eastern European countries where trichinellosis is endemic.
